# A landscape analysis of psychedelic retreat organizations advertising online

**DOI:** 10.1371/journal.pone.0321648

**Published:** 2025-05-02

**Authors:** Logan Neitzke-Spruill, Caroline S. Beit, Jill Oliver Robinson, Nikita Singh, Srijith Kambala, Rishi Ramesh, Amy L. McGuire

**Affiliations:** 1 Baylor College of Medicine, Center for Medical Ethics and Health Policy, Houston, Texas, United States of America; 2 Johns Hopkins University School of Medicine, Baltimore, Maryland, United States of America; 3 Rice University, School of Humanities, Houston, Texas, United States of America; 4 Texas A&M University, School of Engineering Medicine, Houston, Texas, United States of America; University of Brescia: Universita degli Studi di Brescia, ITALY

## Abstract

Research into psychedelics’ clinical potential has corresponded to a growth in public interest and adult use. One common pathway to accessing psychedelics is through psychedelic retreats. While individual retreats have been characterized in the anthropological literature, no systematic evaluation of the psychedelic retreat industry exists. Assessing the characteristics of the psychedelic retreat industry is critical to understanding the associated ethical, legal, and social implications and ensuring consumer safety. To this end, we conducted a landscape analysis of online, publicly available information to capture and characterize a broad range of organizations offering psychedelic retreats and marketing to English-speaking consumers. From July 2023 to December 2023, we identified 298 psychedelic retreat organizations. Some identified as religious organizations, but the majority focused on general wellness. Organizations offered various psychedelic substances with ayahuasca being the most common, followed by psilocybin and San Pedro. Organizations held retreats across the globe at various price points. In total, there were 440 distinct physical locations where retreat experiences were held; 130 were inside the United States (U.S.) and 310 were outside the U.S. Further research into the practices of psychedelic retreat organizations is recommended to help reduce harm and support consumer education.

## Introduction

Psychedelics are a class of naturally occurring and synthetic psychoactive substances grouped for their similar phenomenological profiles and pharmacological action. Several of these substances are used by indigenous groups as part of spiritual, religious and healing practices, and the historical and archeological record indicates psychedelics have been used by humans for millennia [1–5]. In the United States (U.S.) these substances are legally classified as Schedule I, which characterizes them as having a high potential for abuse and no accepted medical use. Recent clinical investigations, however, suggest psychedelics can be effective for treating several psychiatric indications such as post-traumatic stress, depression, anxiety, and substance use disorder [6,7]. Contemporary research has generated substantial public interest in and enthusiasm for psychedelics. It has also led to bipartisan support for psychedelic research and the revitalization of psychedelic culture online and in person [8]. This represents a significant shift from the cultural stigma attached to these substances that contributed to the passage of the Controlled Substances Act in 1970, which effectively enacted a moratorium on psychedelic research that would last more than twenty years.

The resurgence of public interest in psychedelics has led to a corresponding increase in rates of adult use of psychedelic substances such as psilocybin, Lysergic acid diethylamide (LSD), and N,N-Dimethyltryptamine (DMT) [9–13]. Several pathways to access psychedelics that circumvent federal prohibition have grown alongside rates of adult use. First, some state- and local-level legal reform efforts have pursued the de-prioritization of law enforcement around psychedelics and acted to establish state-sanctioned avenues for accessing psychedelic substances [14,15]. Second, so-called “gray markets” have emerged in municipalities with loosened legal restrictions and through digital marketplaces to facilitate access [16,17]. Third, there has been a growth in psychedelic drug tourism and the formation of organizations hosting psychedelic retreats domestically and abroad to meet this demand. The present study addresses this third development by exploring the landscape of psychedelic retreat organizations marketed online to English-speaking consumers.

Psychedelic retreats are central to the phenomenon of drug tourism, which entails tourist experiences involving or specifically aimed at the procurement or consumption of drugs in locales with more permissive or ambiguous drug laws. Prior research on psychedelic retreats has predominately addressed the growth of ayahuasca tourism, which is central to the religious practices of indigenous groups in South America and syncretic new religious movements [18–20]. Due to the cultural significance of ayahuasca and the association of psychedelic effects with spiritual and mystical experience, ayahuasca retreats often draw individuals seeking out spiritual and cultural experiences [21–23]. This has been similarly observed with psilocybin tourism in Mexico [24]. Some retreats, however, offer psychedelics divorced from their original cultural context [25–27].

Motivations for attending psychedelic retreats are consistent with the reasons people report using psychedelics in general, which tend to reflect health, wellness, and enhancement narratives that inform contemporary interest in psychedelics [28,29]. For instance, psychedelic users commonly cite reasons such as management of or coping with mental health ailments, self-improvement or enhancement, or a desire for self-knowledge [30–36]. In addition to health and wellness motivations, spirituality (in line with historical and contemporary associations with religious and mystical experience), and curiosity are common reasons people report using psychedelics [32–34,37]. A recent study of 26 individuals who participated in psychedelic retreats found similar motivations for participation. Personal development and self-medication were the most common motivations, however participants also cited curiosity, peer recommendation, dealing with relationship issues, spirituality, and professionalization for careers in psychedelic therapy as reasons to seek out a psychedelic retreat [38]. Other studies specifically focusing on ayahuasca have found that retreat attendees sought out ayahuasca to treat illness or addiction, attain self-knowledge, grow or develop spiritually, address psychological ailments, deal with personal crises, or achieve “inner healing” [21,22,39]. As the field of psychedelic science continues to expand and public interest in using psychedelics grows, understanding the ways in which people access psychedelics takes on greater importance, especially given the health motivations of many users.

While some retreats have been characterized in the scientific literature [19,21,22,26], the scope and nature of the psychedelic retreat industry remains unknown. This study aims to fill this gap by describing the landscape of psychedelic retreat organizations marketing to English-speaking consumers using online information. Examining psychedelic retreat organizations and their offerings is critical to understanding the associated ethical, legal, and social implications, as well as ensuring the safe and responsible use of psychedelics.

## Materials and methods

### Study design

We conducted a landscape analysis of online, publicly available information to capture and characterize a broad range of organizations that offer psychedelic retreats and market to English-speaking consumers. Data were collected from July 2023 to December 2023. This project received ethics approval from the Baylor College of Medicine Institutional Review Board.

Included organizations met two criteria: 1) marketing materials were in English, and 2) they facilitated psychedelic experiences. Organizations were considered to offer psychedelic retreat services if they facilitated at least one retreat that included the use of psychedelics. We defined a psychedelic retreat as a structured experience around the use of psychedelics provided to either an individual or group. Psychedelics eligible for inclusion encompassed classic psychedelics, such as ayahuasca, psilocybin, lysergic acid diethylamide (LSD), dimethyltryptamine (DMT), mescaline, peyote, and San Pedro, as well as Bufo alvarius venom (5-meo-DMT), ibogaine, Hawaiaka, and 3,4-Methyl enedioxy methamphetamine (MDMA). We also included organizations if their website marketed psychedelic retreats but did not specify the specific substance. Notably, although there are an increasing number of ketamine retreat offerings in the U.S. and abroad, we did not include ketamine offerings given its 1) legal status as a medically approved drug, 2) distinct pharmacological and phenomenological profile, and 3) general exclusion from the category of psychedelics. We excluded organizations that did not offer psychedelic retreats, including those only offering services adjacent to psychedelic use (such as psychedelic integration services) and those solely offering retreats with non-psychedelic substances (for example, essential oils or cacao). Additionally, organizations that did not have functional websites or appeared fraudulent were excluded.

We conducted online searches by entering keywords into Google (see supplement for key terms) and reviewing sources, such as the organization’s website, social media accounts (Instagram, Facebook, YouTube, and TikTok), and relevant news articles, if available, about the organization. Key search terms included: name of psychedelic substances (“ayahuasca,” “psilocybin,” “San Pedro,” “ibogaine,” “bufo,” “Peyote,” “DMT,” “MDMA,” “LSD,” “Mescaline,” “Hawaiaka”), “psychedelic,” or “entheogen” AND “retreat(s),” “center(s),” “experience(s),” “church(es),” “temple(s),” “religion(s),” “medical center(s),” “therapy,” or “organization(s).” Four authors, CSB, NS, RR, and SK, collected and entered data into a REDCap database developed to manage and store the data [40]. Data were reviewed for accuracy during and after data collection by CSB, NS, RR, LNS, and SK. Data collection covered four key domains: 1) operations base and organization type, 2) location of psychedelic retreats, 3) substances offered, and 4) length and cost of psychedelic retreat experiences.

### Data analysis

Descriptive statistics, including frequencies, means, and medians were calculated using Microsoft Excel. Organization types were categorized as wellness, religious, medical, or other based on self-identified characteristics included in online materials. Categories were developed after an initial review of the data. Organizations could be placed into multiple categories. Categorizations were checked for accuracy and consistency and any discrepancies were discussed by the team to reach consensus. Online information about organizations categorized as wellness referred to general notions of “healing,” spotlighted health and wellness activities, lacked connection to any specific religious traditions or referred to non-specific traditions, or contained descriptions of psychedelics’ benefits in terms of personal growth and development. Organizations categorized as religious self-described as a religious organization, church, temple, or as providing religious services and displayed other features of a religion such as providing a statement of beliefs or other religious literature, having non-profit status, or including a membership requirement. Organizations were categorized as medical if they self-described as a medical organization and/or they provided psychedelics explicitly for therapeutic purposes for specific conditions or were employing psychedelics in a psychedelic-assisted therapy (PAT) format. Organizations that self-described as fulfilling a purpose that did not fit the criteria of the preceding three categories were categorized as other.

Organizations’ operations base and retreat locations were categorized by whether they were inside or outside of the U.S. Locations in the U.S. included all 50 states as well as U.S. territories and were further categorized into the four major geographic regions of the Northeast, Midwest, South, and West [41]. Locations outside the U.S. were determined by the continent criteria defined by the World Population Review [42].

The number of different retreat experiences was calculated based on the length of the experience and the substance offered during the experience. For example, if an organization offered a one-day psilocybin retreat, a 4-day psilocybin retreat, and a 4-day ayahuasca retreat, that organization was calculated to have three distinct retreat experiences. It was not possible to calculate the number of times a retreat experience was held because that information was not readily available online.

Costs of an individual retreat experience often varied based upon the accommodations or amenities a participant could choose. As such, we calculated the average cost by length of the retreat experience and based on the minimum and maximum price available online. Currencies were converted as necessary into USD [43]. Costs were calculated based on listed prices, although some organizations indicated that they offered scholarships or other discounts in certain cases, without further details about case eligibility or the amount of these discounts.

## Results

We identified 338 potential retreat organizations. We excluded 40 organizations: 23 did not offer psychedelics, seven did not have fully operational websites, five were duplicates, and five were excluded for other reasons, such as being a general website for an overarching organization, being the webpage of a specific shaman, or being a third-party organization that was not directly organizing retreats. A total of 298 (88.2%) organizations met the above criteria and were included in our analysis.

### Operations base and organization type

**Fig 1:**
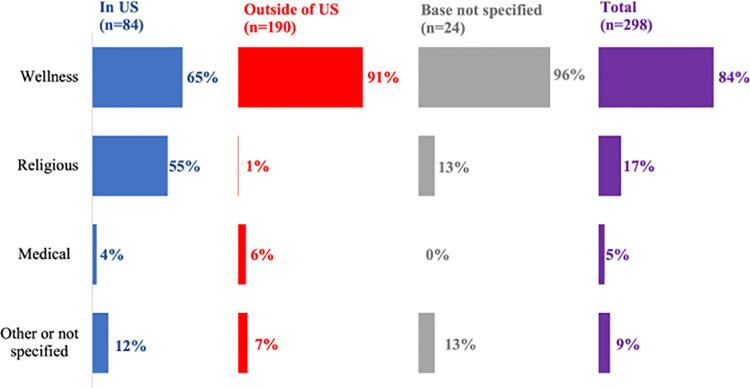
Organization type by location of operations base. Categories are not mutually exclusive. Percentages are based on denominators shown in column headings and may sum to more than 100%.

Most organizations (n=190, 63.8%) had operations bases outside of the U.S.; 84 organizations (28.2%) had operations bases in the U.S., and 24 (8.1%) did not specify the location of their operations base (Fig 1). Based on available online information, 250 (83.9%) were categorized as wellness organizations, 51 (17.1%) were categorized as religious organizations, 15 (5.0%) were categorized as medical, 26 (8.7%) were categorized as other (including identifying as an Ecovillage Project, offering shamanic coaching, or being a mobile ministry, teaching center, or school), and one (0.3%) did not provide enough information to be categorized.

The majority of organizations were categorized as wellness whether based in the U.S. (n=55, 65.5%) or outside the U.S. (n=172, 90.5%). Notably, more than half (n=46, 54.7%) of the 84 organizations based in the U.S. were categorized as religious compared to only 1% of the 190 organizations based outside of the U.S. Only three (3.6%, n=3/84) organizations marketed themselves as medical in the U.S. as compared to twelve (6.3%, n=12/190) organizations based outside the U.S.

Organizations categorized as wellness, the largest category overall, described their offerings in ways consistent with definitions of wellness culture. Organizations in this category portrayed psychedelics as facilitating personal growth or transformation, self-exploration, healing, or spiritual experiences (absent any other features of religion and within a framework of holistic individual well-being). For instance, organizations that offered wellness retreats commonly made statements about how their offerings could help “nurture the natural process of self-realization,” “heal and transform your life,” or “master your mind’s muscle.” Several also invoked ties to indigenous communities and their traditions as a defining feature or selling point. Such organizations commonly emphasized how their offerings were “authentic” or “grounded in ancestral wisdom.”

Organizations categorized as religious were more common in the U.S. than outside of the U.S., comprising 54.7% of all retreat organizations operating in the U.S. These retreat organizations required membership to participate, included “statements of belief” or other religious scriptures, or indicated their religion’s tax-exempt status. Organizations categorized as religious characterized their psychedelic offerings as ritual practices indicated by their religious belief. For example, psychedelic substances were often described as “sacred,” “sacraments,” or “entheogenic.”

Finally, organizations categorized as medical were the least common type of organization identified. These organizations included those that claimed to employ medical techniques or hold medical licensure, used psychedelics for treatment or rehabilitation of specific ailments, or facilitated psychedelic retreats in addition to broader aims of conducting clinical research. For example, one retreat described itself as “a fully-licensed alternative care center.” Organizations offering ibogaine as an addiction treatment featured prominently in this category, though other organizations categorized as medical offered other substances.

### Location of psychedelic retreats

The majority of organizations (69.8%, n=208) offered retreats in only one location, 27.5% (n=82) in more than one location, and 2.7% (n=8) did not provide any information about the locations where they offered retreats. On average, organizations offered retreats at 1.5 (standard deviation =1.2, Max = 10, Min = 1) locations. Three organizations that did not specify a particular retreat location stated that they provided information about the “location upon inquiry,” expressed a willingness to “travel for private retreats,” and offered retreats “globally.”

**Fig 2:**
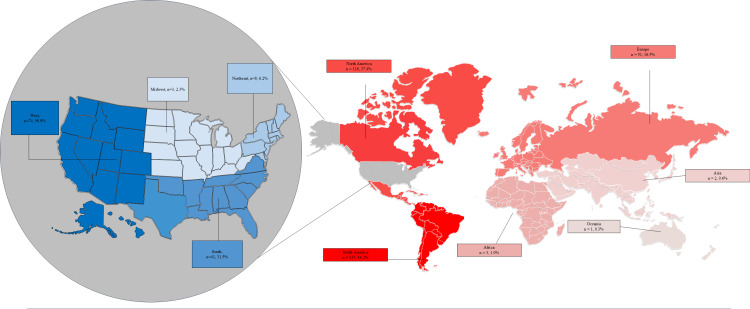
Locations where psychedelic retreats were offered. This map is based on commonly accepted border and continent divisions. (World Population Review, 2024). The total n for retreat locations in the U.S. is 130 and is what the percentages for the U.S. is based on. The total n for retreat locations outside of the U.S. is 310 and is what percentages for the continents are based on. The number of retreat locations in North America does not include retreat locations in the U.S. Additionally, there were 7 online locations, 3 unspecified locations, and 4 locations in the US with the region not specified. Reprinted from SlideEgg.com under CC By license, with permission from Deckzi Solutions Private Limited - SlideEgg, original copyright 2024.

We identified a total of 648 different retreat experiences held in 440 distinct physical locations as well as seven online locations and three other locations (Fig 2). Of the 440 physical locations, a majority (n=246, 55.9%) were somewhere in North America, including 130 (29.5%) locations in the U.S. Most of the 130 locations in the U.S. were in the Western U.S. (n=74/130, 56.9%) and Southern (n=41/130, 31.5%) U.S. regions. Fewer were in the Northeastern (n=8/130, 6.2%) and Midwestern (n=3/130, 2.3%) U.S. regions. Four (3.1%) of the retreat locations in the U.S. did not specify which region they were located in.

Of the 310 physical locations outside of the U.S., 44.2% (n=137/310) were in South America. A few were held in Africa (n=3, 1.0%), Asia (n=2, 0.6%), and Oceania (n=1, 0.3%). Excluding the U.S., North America had the second highest concentration (n=116/310, 37.4%) of retreat locations.

Of note, 23 (27.3%) of the 84 organizations with operations bases in the U.S. had at least one location where they offered retreats outside of the U.S., whereas only five (2.6%) of the 190 organizations with operations bases outside the U.S. had at least one location where they offered retreats inside of the U.S.

### Substances offered

As shown in Table 1, the majority of organizations offered ayahuasca (n=220/298, 73.8%) to their participants, while 76 (25.5%) offered psilocybin and 60 (20.1%) offered San Pedro. The least frequently offered substances were LSD (0.7%), mescaline (0.7%), and Hawaiaka (0.3%). Eight organizations (2.7%) did not specify the psychedelic substances offered. About a third of organizations (n=93, 31.2%) offered more than one substance. Of the 93 organizations that offered more than one substance, 68 organizations specified that they offered more than one substance during at least one retreat experience; 25 specified offering more than one substance across different retreat experiences.

**Table 1:** Substances offered by psychedelic retreat organizations.

Substance	*n*, (%)*
Ayahuasca	220 (73.8)
Psilocybin	76 (25.5)
San Pedro	60 (20.1)
Ibogaine	21 (7.0)
Bufo	20 (6.7)
Peyote	11 (3.7)
DMT	7 (2.3)
MDMA	6 (2.0)
Other (e.g., LSD, mescaline, Hawaiaka, not specified)	13 (4.4)
More than one	93 (31.2)

*Refers to the number of organizations that offered this substance. Percent is out of the total number of organizations n=298. Organizations may have offered more than one substance.

Fifty-six (25.5%) of the 220 organizations that offered ayahuasca were based in the U.S., 150 (68.1%) were based outside of the U.S., and 14 (6.4%) had unspecified locations. Thirty-one (40.8%) of the 76 organizations that offered psilocybin had operations bases in the U.S., 36 (47.4%) outside of the U.S., and 9 (11.8%) had operations bases that were not specified. Eight (13.3%) of the 60 organizations that offered San Pedro had operations bases in the U.S., 50 (83.3%) had bases outside of the U.S., and 2 (3.3%) had unspecified locations.

### Duration and cost of psychedelic retreats

We identified a total of 648 different retreat experiences across the 298 organizations included in our analysis. Each retreat experience may have occurred more than one time; however, we could not calculate how many times each retreat experience was held because that information was not consistently available online. The majority of the 298 organizations (n=159, 53.5%) offered multiple different retreat experiences (average number of experiences = 2.2 per organization, standard deviation = 1.5).

Of the 648 retreat experiences, 589 (90.9%) had a specified duration published online, while 59 (9.1%) did not specify duration. Additionally, 52 (8.0%) retreat experiences had a specified duration that did not fit under our schema (for example, a duration of "a minimum of 6 days”). Retreats that lasted 1–2 weeks were the most common (n=117, 18.1%). Table 2 lists the average price per retreat experience according to retreat duration.

**Table 2:** Retreat duration and cost.

Retreat Duration, (n=648 retreat experiences)	Average Price in USD	Min Price – Max Price in USD
1 day (no overnight), (58)	668	20–3,625
2 days, 1 night, (31)	681	80–5,899
3 days, 2 nights, (77)	980	200–3,333
4 days, 3 nights, (46)	2,218	295–10,000
5 days, 4 nights, (49)	2,914	420–13,500
6 days, 5 nights, (24)	2,696	610–7,238
7 days, 6 nights, (85)	2,665	780–11,250
1–2 weeks, (117)	3,165	825–23,500
Greater than 2 weeks, (50)	7,179	850–150,000

Not all organizations specified the cost of their retreat experiences; 513 (79.2%) of the 648 identified retreat experiences had a specified cost and 135 (20.8%) did not. The price for participating in a retreat ranged from 20 USD – 150,000 USD, with the average cost ranging from 668 USD for one-day retreats to 7,179 USD for retreats that run longer than 2 weeks. Not included in this range was one outlier retreat experience that was greater than 2 weeks and had a maximum price of 500,000 USD.

## Discussion

To our knowledge, this paper is the first to characterize the growing number of psychedelic retreat organizations that market to English-speaking consumers. With 298 organizations offering 648 distinct psychedelic retreat experiences, our study provides an expansive look at the rapidly changing landscape of retreat-based psychedelic tourism amid the growth of scientific knowledge and public interest surrounding these substances [44].

The majority of organizations we identified offered naturally occurring substances and preparations during their retreat(s). Substances such as ayahuasca, psilocybin, and San Pedro were the most common individual substances offered, whereas substances such as MDMA and LSD were relatively infrequent offerings. This is likely because naturally occurring substances are more easily accessible, less subject to legal restrictions, more difficult to detect or identify by law enforcement, and/or used in traditional rites of indigenous groups local to the Americas. Ayahuasca, for instance, was the most commonly offered substance by retreats we identified, despite the fact that it has not been as extensively studied in clinical trials [45–48]. This is unsurprising, however, given the longstanding use of ayahuasca among indigenous groups in Latin America and the proportion of retreats we identified that were based and held in South America. Yet, consistent with the globalization of ayahuasca practices through syncretic religions, neo-shamanism, and Western biomedical interests, we found ayahuasca being offered by retreats globally in locations where the plant constituents of the brew do not occur naturally and no local ayahuasca traditions exist. Additionally, although ayahuasca was by far the most commonly offered substance, the variety of substances being offered by retreat organizations marks shifting demands in the drug tourism market. Specifically, while ayahuasca has historically been the primary draw for psychedelic tourists, the diffusion of knowledge about psychedelics stemming from the psychedelic science movement may contribute to interest in more diverse types of experiences with different drugs. For instance, some may be searching for effects specific to certain drugs, as in the case of individuals with substance use disorder seeking out ibogaine. Alternatively, the use of synthetic psilocybin in clinical trials of PAT may be driving a demand for opportunities to use psilocybin mushrooms in retreat contexts employing similar procedures. Regardless of substance type, presentation of the psychedelics on offer as tools for improving or optimizing health and wellness was common for the retreats we identified.

Consistent with the growth of health and wellness narratives associated with psychedelics [28,29], wellness organizations were the most prevalent in our sample, as well as the most common type of psychedelic organization based outside the U.S. These organizations often presented psychedelics as holistic medicines, incorporated other spiritual or wellness practices (such as yoga, meditation, or reiki) into their retreat offerings, or associated themselves with specific indigenous communities (such as the Shipibo people of the Peruvian Amazon) and their traditions. Religious organizations were considerably less common in our overall sample and were concentrated in the U.S. Organizations that explicitly claimed to employ medical techniques or hold medical licensure, use psychedelics for treatment or rehabilitation of specific ailments, or facilitate psychedelic retreats in addition to broader aims of conducting clinical research were least prevalent in our sample, with only three organizations in the U.S. falling into that category. This is unsurprising given that no psychedelic substance is currently FDA approved for medical use and our focus was on retreat organizations operating outside the healthcare system. However, as psychedelic medicines near approval in the U.S. and public enthusiasm about their therapeutic potential surges, there is an increased risk that individuals will self-treat serious medical conditions by participating in non-medical retreats without appropriate supervision. This may be particularly problematic given that in some cases psychedelics can lead to psychologically destabilizing consequences, such as disorientation or increased sensitivity following the experience, exacerbate mental health conditions, or instigate new symptoms [49]. It is also not clear how psychedelic substances are dosed or administered in the retreat setting (i.e., both the amount of a given substance and the nature and extent of psychological support), and there is only some scientific evidence examining clinical outcomes when psychedelic substances are administered outside standard protocols of PAT [50–56].

Studies of psychedelic retreats suggest that retreat participants perceive several benefits to mental health and well-being from their retreat attendance, which are often attributed to “transformative” changes stemming from psychedelic experiences had during the retreat [26,38,54,55]. The methodology employed here was not geared toward understanding the experiences of retreat participants. However, psychedelic retreats are not without risks, and our analysis highlights several potential concerns.

One issue associated with the growing market for psychedelic retreats is the risk that opportunistic actors lacking sufficient knowledge of psychedelics and how to care for people experiencing their effects will generate harm by providing psychedelics to those who ought to refrain, being unable to help people through challenging experiences, or engaging in other potentially unsafe practices. Several unsafe scenarios may arise to cut costs and maximize profits, such as accommodating more retreat participants than there is staff to handle. Additionally, perhaps in an attempt to cater to retreat participants’ work schedules and limited budgets, some organizations offered one-day or short, weekend retreats. These shortened experiences may increase the likelihood of negative outcomes for participants who become overwhelmed due to the condensed timeline and lack of adequate preparation. Alternatively, should psychologically difficult material emerge during a short psychedelic retreat, participants may require more time to adequately process their experiences before returning to their daily lives. Both preparation and integration are considered important—and time-consuming—components of contemporary PAT [57], which are likely to be overlooked during a retreat lasting only a few days.

Polydrug use is another notable safety concern that emerged in our landscape analysis. Many of the retreats we identified offered more than one drug over the course of a single retreat. Although some polydrug combinations have traditional significance and others may be benign from a safety standpoint [58], there is little research that addresses the safety of mixing psychedelics. Further research is necessary to determine the possible risks and benefits of mixing multiple psychedelics. In the meantime, the potential for retreat organizers to administer multiple substances to cater to retreat participants seeking remarkable experiences raises concerns about both drug interactions and psychological consequences.

We also found that the information presented on retreat organizations’ websites and social media pages—a mixture of marketing and educational material—was often incomplete. Although potential retreat participants could learn more information by contacting retreat organizations directly, disparities in the information available online may hinder retreat participants’ ability to make an informed choice. For example, in our sample, eight organizations did not disclose the specific psychedelic substances they offered, and it was not possible for us to verify if the substances listed online were the only substances offered at the retreat. This approach may have been adopted by these organizations to avoid legal scrutiny or liability given the often-ambiguous legal status of psychedelic retreats in various locations [59]. However, this omission may impede a consumer’s ability to make informed choices when selecting a retreat experience. As decision making regarding psychedelic retreat attendance ultimately falls on consumers, the question of harm-reducing consumer education takes on greater importance.

Future research should seek to identify other domains of information that may be missing on these organizations’ websites and social media pages. Although some researchers, advocacy groups, and retreat organizations have sought to develop ethical guidelines and best practices [60–62], there are currently few reliable markers for identifying trustworthy retreat organizations, nor does there seem to be an adequate understanding of the baseline level of knowledge characteristic of the average psychedelic retreat participant. Some services, such as review platforms like *AyaAdvisors* (https://ayaadvisors.org/), *Retreat.guru* (https://retreat.guru/), and *TheThirdWave* (https://thethirdwave.co/), or psychedelic concierge services, which advise paying customers on which psychedelic retreats to attend based on practices, protocols, and personal networks have emerged to fill this gap [63]. Nonetheless further research geared toward harm reducing education is necessary since these services are subject to bias and can be exceedingly expensive, thereby limiting reach and effectiveness. For instance, while some concierge services are volunteer based, others charge “$10,000 or more for [their] services” with some clients paying “six figures for a consultation” [63].

Clinicians can also play a prominent role in addressing inequity and reducing potential harms associated with unregulated psychedelic use. Given the increased interest in psychedelic retreats, consulting with patients about risks and possible contraindications with preexisting conditions and medications will be important to help them make informed decisions about retreat participation. Therefore, concurrent with educating potential users about important considerations for taking psychedelics on a retreat, further efforts should be made to educate clinicians to prepare them for addressing these questions with their patients. Currently, little is known about clinicians’ knowledge about psychedelic retreats. While the increase in use of psychedelics and potential for clinical approval warrants greater efforts to educate clinicians about psychedelic substances more generally, further research should also explore relevant healthcare professionals’ perceptions of psychedelic retreats and what information they need to appropriately counsel patients seeking this type of experience.

It is likely that psychedelic retreat offerings will continue to expand, even post regulatory approval of psychedelic medicines, given the health-related motivations for many psychedelics users [21,30,32,35,39], these organizations’ appeals to principles of wellness, and especially in light of the projected cost of PAT. Psilocybin assisted therapy is estimated to cost 7747.80–9668.33 USD “depending on the price of psilocybin” [64]. Phase 3 trials of MDMA cost over 11,000 USD per patient [65]. While studies estimating high costs suggest overall savings in terms of added quality of life years, the psychedelic retreat space may allow for supervised use of psychedelics at a comparatively cheaper cost than traditional medical care, with the exception of some retreat offerings that consist of weeks-long stays complete with menus of wellness amenities. In general, the average retreats we found were less expensive and tout similar potential for healing, making them seem more attainable comparably for the average person seeking mental health treatment. Yet, the cost of a psychedelic retreat may still be prohibitive for many people, and if costs remain prohibitive, bad actors may arise offering lower prices with greater risks as demand increases. Inequitable access to psychedelics, which has already been identified as an issue in clinical research [66–68], may be further exacerbated by the potential for the emergence of a two-tiered system based upon an individual’s resources and access to medical care. The portrayal of wellness-oriented retreats as a potentially lower-cost alternative to PAT in clinical settings could further exacerbate health disparities, since retreat settings may be less standardized, riskier, and less likely to employ evidence-based practices.

There are also potential legal risks associated with retreat participation that consumers should be aware of. Many of the countries where retreats are being held prohibit the use of psychedelics with some exceptions for research and religious practices. For example, 28.2% of retreat organizations that we identified were based in the U.S. and 29.5% offered retreats at a location in the U.S., even though all classic psychedelics are federally illegal under the U.S. Controlled Substances Act. Some are willing to assume these risks, while others seek to avoid them by traveling to countries with long traditions of psychedelic use and more permissive drug policies [69,70]. This trend is reflected in the number of retreats we identified that were held in South America, which accounted for 44.2% of all international locations in our sample. For consumers, traveling to South America for a psychedelic retreat may be more expensive, but it can afford them greater legal protection and may also provide the impression that they are receiving a more “authentic experience” in a traditional context, which research suggests some consumers value [20,22,69,71].

Finally, the proliferation of psychedelic retreats and the demand for “authentic” indigenous healing experiences potentiates broader social and ecological harms. Specifically, there are several implications associated with the commodification of plants used as traditional sacraments and medicines by indigenous groups, such as ayahuasca, San Pedro, and peyote. Several have raised concerns over the commercialization of indigenous culture and the need for reparations and reciprocity with the communities whose practices are central to the offerings of retreat organizations [72–75]. This is particularly a concern given assumptions about foreign ownership of retreat organizations operating in Latin American countries that utilize local labor for shamanic ceremonies and accommodations for psychedelic tourists. Similar to other instances of commercial interest in natural resources that exist in the homelands of indigenous people, the demand can create community-level conflict over whether to help facilitate or participate in an extractive industry. Future research should continue to critically examine these issues, including indigenous perspectives on psychedelic tourism and the potential for cultural appropriation when ceremonial practices are integrated into retreats that are facilitated by non-indigenous practitioners.

## Limitations

This research is subject to several limitations. First, our compiled list of 298 psychedelic retreat organizations, though substantial, may not encompass all organizations and the information may change or become obsolete over time. Second, the data we collected were based on publicly available, online information, which can be incomplete or inaccurate, especially given the dynamic and growing nature of the psychedelic retreat landscape. Most companies provided limited descriptive information about their retreat offerings, which did not allow for more in-depth analysis of other key dimensions, such as staff-to-participant ratio or adoption of specific safety protocols. This information is important and should be made more easily accessible to prospective consumers. However, the completeness and accuracy of the information that is made available may be impacted by the varying legal status and ramifications of psychedelics, especially in locations with stringent laws. While we reviewed the data collected for accuracy, additional research to gather more complete information and verify the accuracy of publicly available information is warranted. Future research should also focus on collecting systematic information about long-term outcomes of retreat participation, including lasting benefits and adverse events. Despite these limitations, the comprehensive and expansive nature of our landscape analysis serves as a valuable overview of the current status of psychedelic retreat organizations and offers essential insights for both consumers and researchers.

## Conclusion

Our analysis of 298 psychedelic retreat organizations provides a powerful and comprehensive overview of the psychedelic retreat landscape, especially as it markets to English speaking consumers. This paper highlights the importance of harm reduction in an unregulated drug market. While many organizations provide essential information online, significant gaps hinder consumers’ ability to make informed decisions. As the psychedelic retreat industry continues to grow, further monitoring is essential to ensure the safety of retreat participants and the sustainability of the industry.
